# Synergistic antimicrobial activity of essential oils mixture of *Moringa oleifera*, *Cinnamomum verum* and *Nigella sativa* against *Staphylococcus aureus* using L-optimal mixture design

**DOI:** 10.1186/s13568-024-01797-y

**Published:** 2025-01-28

**Authors:** Samah H. Abu-Hussien, Antony R. Nasry, Ziad Samy, Salwa M. El-Sayed, Ashraf Bakry, Naglaa Ebeed, Hesham Elhariry, Thanaa ElNoby

**Affiliations:** 1 Department of Agricultural Microbiology, Faculty of Agriculture, Ain Shams University, Cairo, 11241 Egypt; 2 Biotechnology Program, Faculty of Agriculture, Ain Shams University, Cairo, 11241 Egypt; 3 Department of Biochemistry, Faculty of Agriculture, Ain Shams University, Cairo, 11241 Egypt; 4 Department of Genetics, Faculty of Agriculture, Ain Shams University, Cairo, 11241 Egypt; 5 Department of Food Science, Faculty of Agriculture, Ain Shams University, Cairo, 11241 Egypt; 6 Department of Agriculture Economics, Faculty of Agriculture, Ain Shams University, Cairo, 11241 Egypt

**Keywords:** Response surface methodology, Combinatorial synergism, Phytochemicals, Chromatography, Cytotoxicity, *Moringa oleifera*, *Cinnamomum verum*, *Nigella sativa*

## Abstract

The urgent need to address the growing problem of antimicrobial resistance in multidrug-resistant bacteria requires the development of pioneering approaches to treatment. The present study aims to evaluate the antimicrobial potential of the essential oils (EOs) of *Moringa oleifera* (moringa), *Cinnamomum verum* (cinnamon), and *Nigella sativa* (black seed) and the synergistic effect of the mixture of these oils against *Staphylococcus aureus *MCC 1351. Statistical modeling revealed cinnamon oil had the highest individual antimicrobial potency, followed by black seed oil. The combination of the three EOs exhibited significant synergistic effects compared to the individual oils, with a Fractional Inhibitory Concentration (∑FIC) index of 0.27. L-Optimal mixture design of response surface methodology (RSM) identified the optimal mixture as moringa: cinnamon: black seed oils by the ratio of (1:1:1) in run 15 (0.338:0.307:0.355 mL) (v/v). This mixture exhibited significant antibacterial efficacy, outperforming individual oils and conventional antibiotics like tetracycline. Specifically, the combination reduced the MIC values from 3.12, 0.78, and 6.25 to 0.25, 0.06, and 0.78 μg/mL for moringa, cinnamon, and black seed oil, respectively. Synergistic interactions between oils further boosted efficacy, with moringa-cinnamon and cinnamon-black seed pairings exhibiting the strongest synergies. The developed predictive models for IZD and MIC showed excellent fit, with R^2^ values of 0.9843 and 0.9958, respectively. Pareto chart analysis highlighted the predominant individual and synergistic effects, with the Moringa-Cinnamon interaction exhibiting the highest positive synergy. Notably, the oil mixture of run 15 demonstrated excellent biocompatibility, maintaining 97.6% viability of normal human skin fibroblasts (HSF) after 24 h exposure to 200 μL EOs of the mixture per mL. Gas chromatography mass spectrometry (GC/MS) identified abundant bioactive phytochemicals like cinnamaldehyde, linoleic acid, and palmitic acid methyl esters underlying the observed antimicrobial effects. This rationally designed, synergistic phytochemical combination presents a promising natural therapeutic against antibiotic-resistant *S. aureus* while exhibiting minimal cytotoxicity. The results underscore how combining essential oils could help address the issue of antibiotic resistance in *S. aureus*.

**Supplementary Information:**

The online version contains supplementary material available at 10.1186/s13568-024-01797-y.

## Introduction

Antimicrobial resistance poses a serious threat to global public health. It is driven by the overuse of antibiotics which leads to resistant strains of bacteria. Common infectious agents such as *S. aureus* exhibit widespread resistance to standard antibiotic therapies (Huemer et al. 2020). Additionally*, S. aureus* and other microbes use biofilm formation to evade antibiotics and immune responses. There is an urgent need for alternative antimicrobial approaches that can overcome resistance mechanisms (Aslan and Akova 2022). Medicinal plants demonstrate promising antibacterial and antibiofilm capabilities and have long been invaluable for traditional medical systems.

*Moringa oleifera*, *Cinnamon verum* (cinnamon), and *Nigella sativa* (black seed) exhibit broad spectrum activity attributed to their diversity of potent phytochemicals that can synergize for enhanced therapeutic effect (Tiwari et al. 2018). Moringa is an exceptionally nutritious tree native to Northern India. Almost all parts of the plant have medicinal uses that have been exploited for centuries. Phytochemicals like quercetin and kaempferol confer confirmed antibacterial action against drug-resistant *S. aureus* and other pathogenic strains (Morgan et al. 2020). Cinnamon, derived from the inner bark of Cinnamomum trees, is a commonly used spice that contains cinnamaldehyde as its primary bioactive constituent. This compound demonstrates confirmed antibacterial, antifungal, antiviral, and anti-parasitic properties against a variety of pathogens (Pereira et al. 2021). The ability of cinnamaldehyde to inhibit cell wall synthesis makes it effective against Gram-positive and Gram-negative bacteria, including *S. aureus* (Doyle et al. 2019). Research indicates synergistic enhancement of antimicrobial potency when cinnamaldehyde is combined with other cinnamon phytochemicals such as eugenol. Beyond bactericidal effects, cinnamon extracts potently eradicate biofilms and prevent biofilm formation in problematic pathogens at levels comparable to standard antibiotics (Esposito and Turku 2023). Antimicrobial efficacy, synergy potential, and biofilm inhibitory activity make cinnamon a promising natural alternative to traditional antibacterial agents (Abd El-Aziz et al. 2021; Vasconcelos et al. 2018). The Black seed’s chief bioactive component thymoquinone shows similar antimicrobial effects, inhibiting *S. aureus* growth through disruption of cell membrane integrity (Adegbeye et al. 2020). Synergism between thymoquinone and other seed phytochemicals likely enhances this action (Dera et al. 2021). While individual plants and isolated compounds exhibit antimicrobial effects, combining phytochemically rich natural extracts provides opportunities for synergistic interactions that heighten therapeutic outcomes (Rahman et al. 2021). The mixture of plants may also suppress microbial resistance mechanisms from operating (Basavegowda and Baek 2022; Kumar and Pal 2018).

Despite demonstrated individual efficacy, few studies have explored the antibacterial and antibiofilm potential of the extract’s mixture of moringa, mint, and black seed in resistant *S. aureus*. Characterizing optimal combinations is key to maximizing the synergy between hundreds of potent phytochemicals these herbs supply. Response surface methodology (RSM) employs statistical experimental design techniques to model and optimize complex processes (Gamal et al. 2020). Mixture design is ideal for optimizing multi-component herbal formulations by evaluating compositional and interaction effects on responses like antibacterial action (Baj et al. 2023). Therefore, this work aims to explore the individual and combinatorial efficacy of *Moringa oleifera*, *Cinnamon verum,* and *Nigella sativa* leaf extracts against *S. aureus* proliferation. The mixture design of RSM will be utilized to characterize this phytochemically rich mixture’s synergistic antibacterial potential as an alternative natural therapeutic against this problematic microbe.

## Materials and methods

### Essential oils

The essential oils of moringa, black seed, and cinnamon were obtained from the National Research Center (NRC), in Cairo, Egypt.

### Bacterial strain

*S. aureus* isolate was obtained from a previous study (Abu-Hussien et al. 2024) and deposited in EMCC culture collection at Microbial resources center (MIRCEN), Cairo, Egypt as *S. aureus* EMCC 1351 and deposited in Genbank with gene accession number OQ766965.

The isolate was maintained at the biology lab, New Programs administration, Faculty of Agriculture, Ain Shams University, Cairo, Egypt at 4 °C and sub-cultured on nutrient agar slants before use.

### Standard inoculum

A conical flask (250 mL) containing 50 mL of nutrient broth was inoculated with a loop of *S. aureus* EMCC 1351. All flasks were incubated on a rotary shaker (150 rpm) for 24 h at 30 °C. The standard inoculum contained (2.5–2.7 × 10^5^ CFU/mL).

### Evaluation of *S. aureus* EMCC 1351 susceptibility to commercial antibiotics

Ten widely used commercial antibiotics were tested against *S. aureus* EMCC 1351. These included amoxicillin (10 µg/disc), norfloxacin (10 µg/disc), doxycycline (30 µg/disc), ampicillin (10 µg/disc), ciprofloxacin (5 µg/disc), azithromycin (15 µg/disc), gentamicin (10 µg/disc), rifampin (5 µg/disc), and tetracycline (10 µg/disc), all sourced from Amoun Company, Cairo, Egypt. One milliliter of each bacterial inoculum (10^6^ CFU/mL) was spread on sterile petri dishes containing Mueller–Hinton Agar (MHA). The 10 antibiotic discs were placed on the center of inoculated plates and incubated at 37 °C for 24 h (Humphries et al. 2018). The antibiotic susceptibility of the tested *S. aureus* was estimated by inhibition zone formation (cm) and categorized as sensitive (S), intermediate (I), and resistant (R) according to the Clinical Laboratory Standard Committee (Humphries et al. 2018). All experiments were carried out in triplicates.

### Antimicrobial potential and minimum inhibitory concentration (MIC) for the EOs against *S. aureus* EMCC 1351

For antimicrobial activity, the agar well diffusion method was applied. Briefly, 500 μL of the tested *S. aureus* strain (10^6^ CFU/mL) were streaked on the surface of Mueller Hinton Agar (MHA) plates. Using a 6 mm diameter cork porer, wells were made on the Petri dishes filled with different concentrations of EOs and then incubated at 37 °C for 24 h. After this incubation period, Inhibition zone diameter (cm) was measured and MIC was tested using microdilution methods (Humphries et al. 2018). Briefly, 500 μL of the tested *S. aureus* strain (10^6^ CFU/mL) were inoculated into TSB supplemented with different concentrations of oil mixture (0.5, 0.25, 0.125, 0.0625, 0.03125 (µg/mL) and then incubated at 37 °C for 24 h. After this incubation period, OD at 620nm was measured. MIC evaluates the lowest level of antimicrobial agent that maximally inhibits 90% of the growth.

### Mixture design for the optimization of EOs mixture against *S. aureus* inhibition

As shown in Table 1 the l-optimal mixture design of RSM was used to calculate all mixture constitutions’ levels. The Sum of all mixture constitutes was expressed mathematically as 0.000 ≤ A: Moringa ≤ 0.660, 0.000 ≤ B: Cinnamon ≤ 0.660, 0.000 ≤ C: Black seed ≤ 0.660, A+B+C =1.000, in which this relationship is called the fundamental constraint of mixtures. An optimal design with a full experiment of 16 runs was chosen for the optimization process as presented in Table 1. The 3D triangle was designed to have the three diluted essential oils located in the mixture of essential oils, the equal portions mixture of the three components at the triangle’s vertices. Run trial No. (2,3,6,7) were replicated 2 times to detect the pure error and to compare it with the lack of fit. Cubic and quadratic models were used to express the responses as a function of independent variables based on the mixture design method in which, Y: The response (inhibition zone diameter) in cm. α1, α2, α3 represent the linear term coefficients. α12, α22, and α23 represent the binary term coefficients. α123 was for the ternary term coefficient (Baj et al. 2023).

**Table 1:** Matrix for the mixture design for EOs combination

Run	Moringa	Cinnamon	Black seed
1	0.660	0.238	0.102
2	0.541	0.459	0.000
3	0.338	0.307	0.355
4	0.194	0.207	0.599
5	0.340	0.000	0.660
6	0.124	0.660	0.216
7	0.000	0.385	0.615
8	0.660	0.050	0.290
9	0.124	0.660	0.216
10	0.136	0.464	0.400
11	0.338	0.307	0.355
12	0.541	0.459	0.000
13	0.459	0.086	0.455
14	0.000	0.385	0.615
15	0.338	0.307	0.355
16	0.340	0.660	0.000

### Synergistic activity for the EOs mixture

For the assessment of the synergistic effects of three essential oils against *S. aureus* EMCC 1351, a combination study using the checkerboard method was conducted (Costa et al. 2019). Sixteen different combinations ofoils were prepared, and the MIC for each combination was determined (Jain 2011). The MIC values were compared to the MIC of each oil alone (Meletiadis et al. 2010). The synergistic interaction was quantified using the Fractional Inhibitory Concentration Index (FICI), calculated as the following equation:

1 $$\text{FICI}=\text{FIC of oil A}+\text{FIC of oil B}+\text{FIC of oil C}$$

where the FIC of each oil is defined as the following equation:

2 $$\text{FIC of oil }=\frac{\text{Concentration of oil in combination}}{\text{MIC of oil alone}}$$

A FICI ≤ 0.5 indicates synergy, 0.5 < FICI ≤ 1 indicates an additive effect, 1 < FICI ≤ 4 indicates indifference, and FICI > 4 indicates antagonism. Additionally, the percentage of synergistic effect was calculated for each combination using the following formula (Costa et al. 2019; Meletiadis et al. 2010):

3 $$\begin{aligned} &{\text{Synergistic effect }}\left( {\% } \right) \\ &\quad\quad = \frac{{{\text{MIC predicted for run}} - {\text{MIC observed for run}}}}{{\text{MIC predicted for run}}} \times 100\end{aligned} $$

### Cytotoxicity of EOs mixture against normal HSF cell line

For detecting the biocompatibility and safety of the active compounds of the EOs mixture before application in human consumption, the cytotoxic activity was investigated in Nawah-Scientific, Cairo, Egypt, using the Oral epithelial cell line (OEC). The cells were grown in Dulbecco's Modified Eagle medium (DMEM), containing 10% Fetal bovine serum (FBS), penicillin (100 units/mL), and streptomycin (100 mg/mL). The cultures were maintained at 37 °C in a humidified atmosphere with 5% CO_2_. Ten concentrations of twofold serially diluted MOL were prepared. The MTT (3-(4,5-Dimethylthiazol-2-yl)-2,5-Diphenyltetrazolium bromide) assay was conducted about (Abu-Hussien et al. 2022), where confluent monolayers of OEC were cultured for 24 h in a 96 well-microtiter plate. The cells were cultured in triplicates with varying doses of the tested MOL at 37 °C for 72 h under a CO_2_ environment. After incubation, 20 µl of 5 mg\mL MTT was gently added to each well, and incubated at 37 °C. After 4 h, the medium was gently removed and then 150 μl MTT solution was added. The plate was covered with tin foil and then the cells were incubated for 15 min. on an orbital shaker (Benchmark Scientific-BT 300, California). Finally, the optical density (OD) was measured at 570 nm in a micro-plate reader (BMGLABTECH®FLUOstar Omega, Germany). A dose distribution curve was constructed using the data obtained after exposing the OEC cell lines to different concentrations of *P. americana* hemolymph.

### Gas chromatography Mass Spectrometry(GC/MS) for the EOs mixture

GC/MS was done to detect the different chemical active compounds in the studied extract. Essential oils were dissolved in methanol solvent. Gas chromatography paired with mass spectrometry was utilized to analyze the chemical components. A capillary column was employed to separate the complex mixture. The column temperature was initially held at 50 °C, then increased at a steady rate up to 250 °C, held briefly, then ramped up further to 300 °C. Helium carrier gas transported the vaporized samples through the column. An auto-injector introduced small sample volumes into a heated inlet, where they were vaporized for analysis. The instrument detected the separated chemicals as they eluted from the column, collecting mass spectra. These spectra were compared to library databases to identify the specific compounds based on fragmentation patterns unique to their structures. Operating parameters for the temperature, flow rate, injection volume, etc. were methodically chosen and controlled. Thus, the system and methodology enabled the characterization of the essential oil constituents (Abu-Hussien et al. 2022).

### Statistical analysis

Data were analyzed by one-way ANOVA followed by Tukey's post-hoc test using SPSS 12. *P* < 0.05 was considered statistically significant. All samples and collected data were statistically analyzed using Design Expert 12 Statistics software (link). A Tukey test at a P-value of 0.05 was applied.

## Results

### Antibiotic potential of commercial antibiotics against *S. aureus* EMCC 1351

**Fig. 1:**
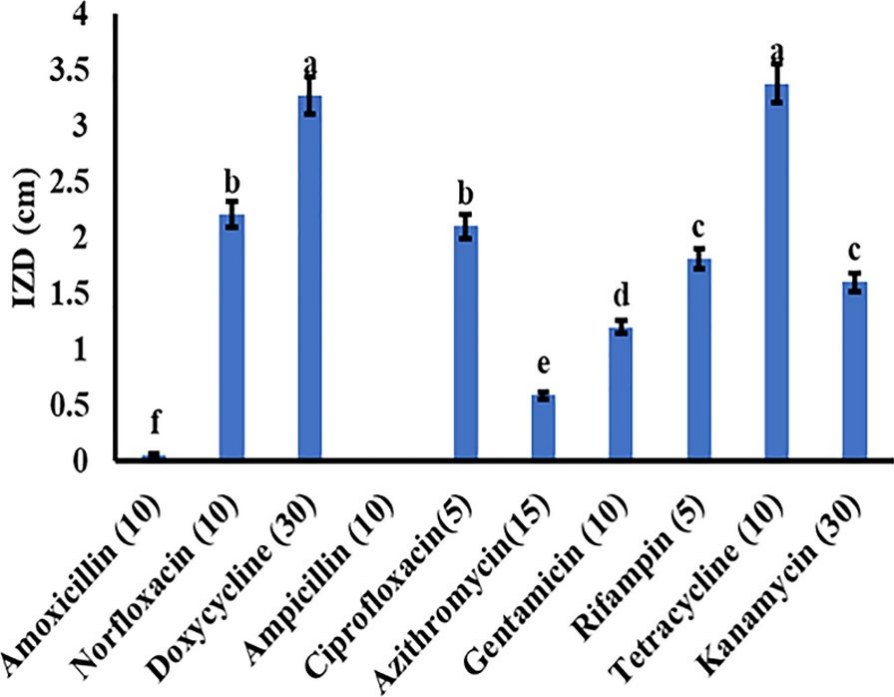
Antibiotic susceptibility test against the tested bacterial pathogenic *S.aureus* EMCC 1351

As shown in Fig. 1, *S. aureus* was strongly sensitive to tetracycline (10 µg/disc) and doxycycline (30 µg/disc) with inhibition zone diameters of 3.38 and 3.27 cm, respectively. While it was resistant to amoxicillin (10 µg/disc), ampicillin (10 µg/disc), and azithromycin (15 µg/disc).

### Antimicrobial activity of EOs mixture against *S. aureus* EMCC 1351

#### MIC of EOs mixture

The antimicrobial interactions between moringa, cinnamon, and black seed oils were evaluated using their individual and combined MIC values as shown in Table 2. The combination of moringa and cinnamon reduced their respective MICs from 3.12 µg/mL and 0.78 µg/mL when tested individually, to 0.63 µg/mL and 0.19 µg/mL, respectively, showing an additive interaction with a ∑FIC index of 0.6. Similarly, the combination of Moringa and Black Seed reduced the MICs to 0.78 µg/mL and 3.12 µg/mL, also displaying an additive effect with a ∑FIC index of 0.74. The combination of cinnamon and black Seed demonstrated a more pronounced additive interaction, with respective MICs of 0.11 µg/mL and 3.12 µg/mL, and a ∑FIC index of 0.63. The strongest result came from the combination of Moringa, Cinnamon, and Black Seed oils, which reduced the MIC values to 0.25 µg/mL, 0.06 µg/mL, and 0.78 µg/mL, respectively, yielding a ∑FIC index of 0.27, indicating strong synergy. These initial findings provided insights into the potential synergistic and additive interactions between these essential oils. To further explore the relationships and refine the optimization of these combinations, a mixture design experiment was conducted with 16 runs, allowing for a more comprehensive understanding of the antimicrobial effects at various concentration ratios of the oils.

**Table 2:** Synergistic antimicrobial interactions of essential oil combinations against *Staphylococcus aureus* EMCC1351

Combination	MIC (µg/mL)		∑FIC	Index	Antibacterial interaction
	Single	Combined			
Moringa/Cinnamon	3.12/0.78	0.63/0.19	0.2/0.24	0.60	Additive
Moringa/Black seed	3.12/6.25	0.78/3.12	0.25/0.49	0.74	Additive
Cinnamon/Black seed	0.78/6.25	0.11/3.12	0.14/0.49	0.63	Additive
Moringa/Cinnamon/Black seed	3.12/0.78/6.25	0.25/0.06/0.78	0.08/0.07/0.12	0.27	Strong synergy

#### Mixture design for optimizing *S. aureus* EMCC 1351 inhibition using EOs mixture

As shown in Table 3, The table presents the experimental design matrix with the observed and predicted values for *S. aureus* inhibition in terms of inhibition zone diameter (IZD) and MIC using the essential oil mixture of moringa, cinnamon, and black seed. The data shows a wide range of IZD values, from as low as 0.5 cm to as high as 5.2 cm, with the observed values closely matching the predicted values. Similarly, the observed MIC values ranged from 0.03125 μg/mL to 0.5 μg/mL, again aligning well with the predicted MIC values.

**Table 3:** Experimental design matrix with the experimental and predicted values for *S. aureus* inhibition IZD (cm) and MIC (µg/mL) and synergy effect (%) using moringa, cinnamon, and black seed essential oil mixture

Run No	Moringa oil	Cinnamon oil	Black seed oil	Observed IZD (cm)	Predicted IZD (cm)	Observed MIC (µg/mL)	Predicted MIC (µg/mL)	Synergy effect (%)	Interpretation
1	0.66	0.238	0.102	3.75	3.74	0.125	0.1173	− 6.5	Antagonism
2	0.541	0.459	0	2.14	2.15	0.25	0.254	1.5	Synergy
3	0.338	0.307	0.355	4.49	4.96	0.0625	0.042	− 48.8	Antagonism
4	0.194	0.207	0.599	4.45	4.46	0.0625	0.069	9.42	Synergy
5	0.34	0	0.66	0.5	0.4969	0.5	0.4977	− 0.46	Antagonism
6	0.124	0.66	0.216	3.42	3.42	0.125	0.1276	2.03	Synergy
7	0	0.385	0.615	3.35	3.35	0.125	0.1244	− 0.48	Indifferent
8	0.66	0.05	0.29	4.04	4.05	0.03125	0.0363	13.91	Synergy
9	0.124	0.66	0.216	3.42	3.42	0.125	0.1276	2.03	Synergy
10	0.136	0.464	0.4	4.26	4.25	0.0625	0.0564	− 10.81	Antagonism
11	0.338	0.307	0.355	5.2	4.96	0.03125	0.042	25.59	Strong synergy
12	0.541	0.459	0	2.14	2.15	0.25	0.254	1.57	Synergy
13	0.459	0.086	0.455	3.76	3.76	0.0625	0.0596	− 4.86	Antagonism
14	0	0.385	0.615	3.35	3.35	0.125	0.1244	− 0.48	Indifferent
15	0.338	0.307	0.355	5.2	4.96	0.03125	0.042	25.59	Strong synergy
16	0.34	0.66	0	3.32	3.31	0.125	0.1196	− 4.51	Antagonism

The table also includes the calculated synergy effect (%) for each run, which indicates the degree of interaction between the essential oil components. Several runs demonstrated synergistic effects, with positive synergy percentages, such as Run 2 (1.5% synergy), Run 4 (9.42% synergy), Run 8 (13.91% synergy), Run 11 (25.59% strong synergy), and Run 12 (1.57% synergy). Conversely, some runs exhibited antagonistic effects, with negative synergy percentages, such as Run 1 (− 6.5% antagonism), Run 3 (− 48.8% antagonism), Run 5 (− 0.46% antagonism), and Run 10 (− 10.81% antagonism). Additionally, a few runs were classified as indifferent, with negligible synergy effects near 0%. These results highlight the varied interactions between the essential oil components and their impact on the overall antimicrobial activity against *S. aureus*.

### Analysis of varience for the EOs mixture against *S. aureus* EMCC 1351

As shown in Table S1 and Table S2, as supplementary materials the cubic and quadratic models developed were statistically significant for *S. aureus* inhibition in terms of IZD and MIC. For IZD, Table 3 shows that the p-value is less than 0.05, showing that the factors had a significant effect. The significant model terms were the interaction between moringa and cinnamon oils (AB), the three-component interaction (ABC), and the interaction between the difference in cinnamon and black seed oil concentrations (BC (B-C)). The F value of 156.76 and Prob > F value less than 0.0500 show that the model is significant for *S. aureus* inhibition. The low Prob > F value (less than 0.05) with a coefficient of determination (R^2^ = 0.984), the standard deviation was 0.2368 cm, and the mean IZD was 3.55 cm. The R-squared values were high at 0.9843 for R^2^, 0.9607 for adjusted R^2^, and 0.9623 for predicted R^2^, again indicating the model fit the experimental data well. The coefficient of variation was 6.67%. Overall, the ANOVA analysis showed that the predictive model had statistically significant terms related to moringa-cinnamon, three-component, and cinnamon-black seed differential interactions. The model fit metrics were very good, confirming the model effectively described how the essential oil components interacted to produce the experimentally observed inhibition zones. Shows that the model terms had a significant effect on the response. In summary, moringa, cinnamon, and black seed essential oils were found to have a significant impact on *S. aureus* inhibition according to the mathematical model developed as shown in terms of coded factors in Eq. (4): Coded equation.

4 $$\begin{aligned} {\text{Y}}_{{{\text{IZD}}}} &= \, 0.{5}0{6592}\, {\text{A }} + \, - {1}.{66948} \,{\text{B }} + \, - {5}.{15}00{5} \,{\text{C }}\\&\quad + { 13}.0{83} \,{\text{AB }} + { 2}0.{6224} \,{\text{AC }} + { 3}0.0{23}\, {\text{BC}} \end{aligned} $$

For MIC, Table S2 shows that the* p*-value is less than 0.05, showing that the factors had a significant effect. The F value of 41.75 and Prob > F value less than 0.0500 show that the model is significant for *S. aureus* inhibition. The low Prob > F value (less than 0.05). The standard deviation was small at 0.0123. R-squared metrics were very high (R^2^ = 0.9958; adjusted R^2^ = 0.9894), and the model terms had a significant effect on the response. The coefficient of variation was 9.38% based on a mean MIC of 0.1309. Significant model terms included the linear mixture components, interactions between moringa-cinnamon (AB), moringa-black seed (AC), cinnamon-black seed (BC), and the difference in cinnamon and black seed concentrations (BC(B-C)). Non-significant terms were the three-component interaction (ABC) and moringa-cinnamon differential (AB(A-B)) interaction. In summary, moringa, cinnamon and black seed essential oils were found to have a significant impact on *S. aureus* inhibition according to the mathematical model developed as shown in terms of coded factors in Eq. (5): Coded equation:

5 $$\begin{aligned} {\text{Y}}_{{{\text{IZD}}}} &= \, 0.{195732}\, {\text{A }} + \, 0.{475697} \,{\text{B }} + \, 0.{963636} \,{\text{C }} + \, - 0.{495452} \,{\text{AB }} \\&\quad+ \, - {1}.{49256} \,{\text{AC }} + \, - {2}.{696}0{4} \,{\text{BC}} \end{aligned}$$

### Antimicrobial potency and synergistic interactions by EOs mixture against *S. aureus* EMCC 1351

**Fig. 2:**
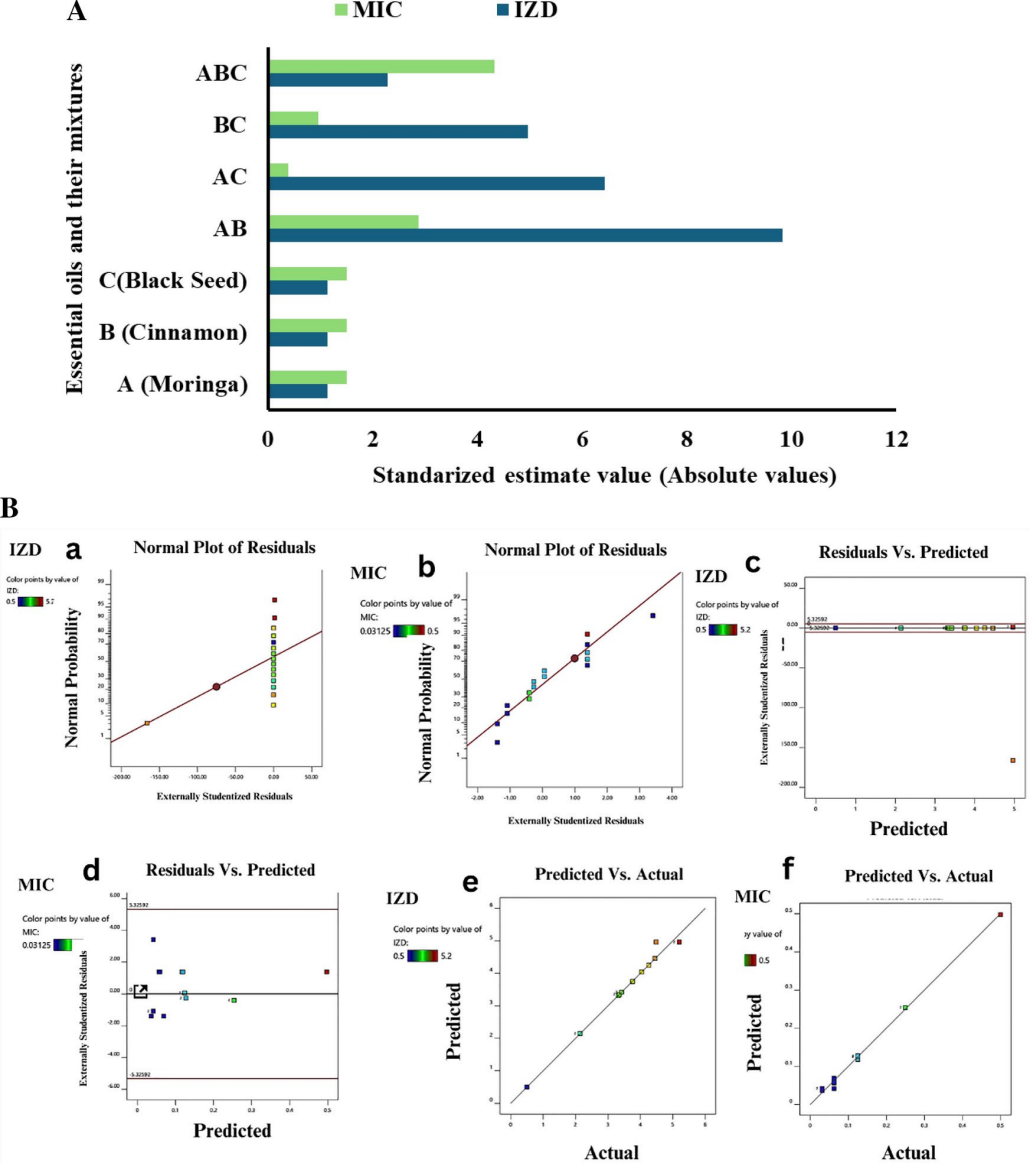

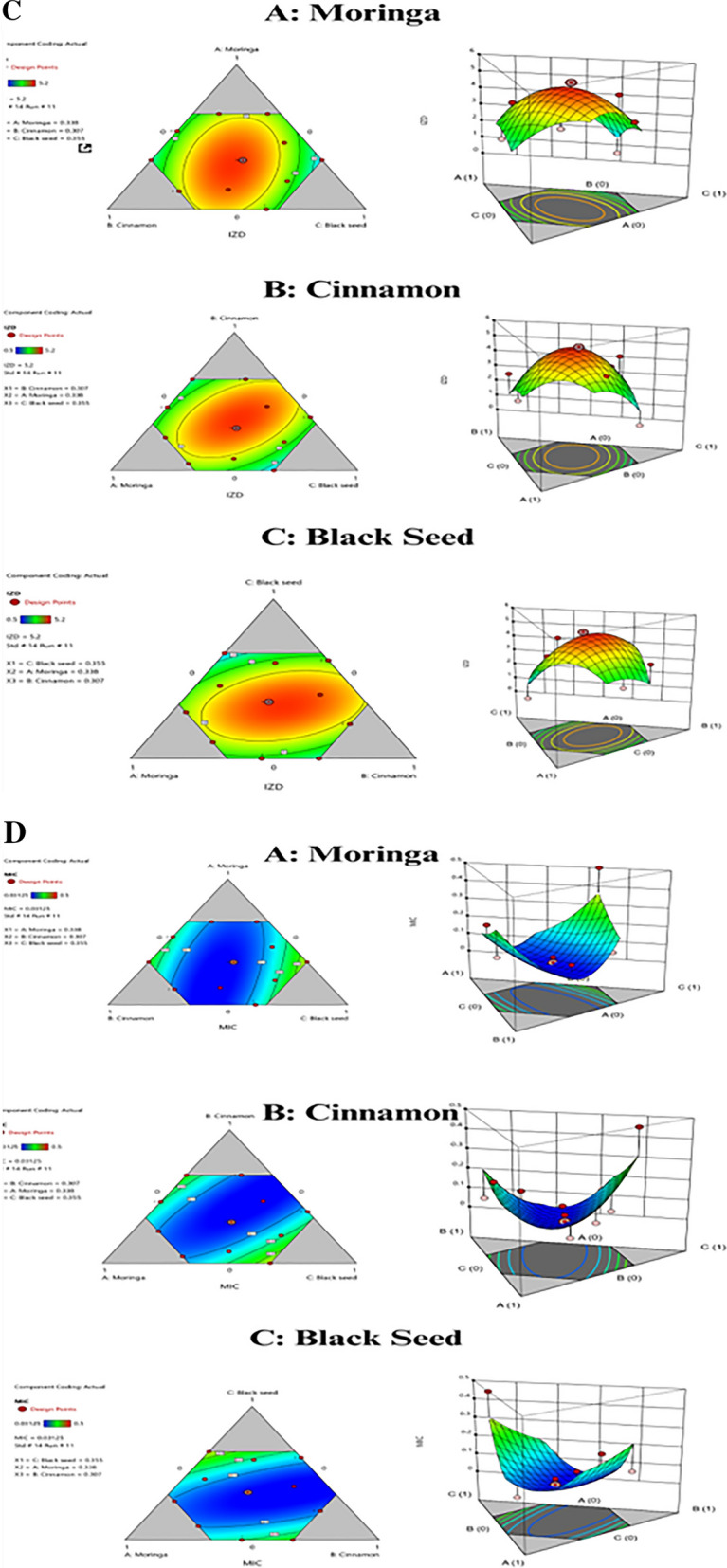
**A** Main ranked significant factors affecting optimal antimicrobial activity of EOs against *S. aureus*.** B** Plot of the predicted values versus the observed values, studentized residuals, and normal percentage of probability of *S. aureus* inhibition efficiency (%) using moringa, cinnamon, and black seed essential oil mixture. **C** 3D surface and 2D contour plot for the interactive factors of *S. aureus* inhibition zone diameter (cm) using moringa, cinnamon, and black seed essential oil mixture. **D** 3D surface and 2D contour plot for the interactive factors of *S. aureus* MIC using moringa, cinnamon, and black seed essential oil mixture

Figure 2A and B show that the standardized estimate values for the individual and interactive effects of the essential oil components (moringa, cinnamon, and black Seed) on the antibacterial activity against *Staphylococcus aureus*, as measured by MIC and inhibition zone diameter (IZD). The data indicates that the individual effect of cinnamon oil (component B) had the highest impact on improving antimicrobial potency, with standardized estimate values of around 16.44 for both MIC and IZD. This was followed by the individual effect of black Seed oil (component C), with values around 15.75. The moringa oil (component A) had the smallest individual effect. In terms of the interactive effects, the combination of moringa and cinnamon oils (AB interaction) showed the strongest synergy, with standardized estimate values of 21.22 for both MIC and IZD. This synergistic boost from the moringa-cinnamon pairing exceeded the individual contributions of the two components. The interaction between cinnamon and black Seed differential (BC(B-C)) also significantly improved the antimicrobial efficacy, with standardized estimate values around 15.09.

The ANOVA results showed that the quadratic model is adequate for predicting *S. aureus* inhibition in the variables studied range. A normal probability plot is used to check the normality distribution of the residuals. Great deviation from normality was not observed in the normal probability plots of the residuals.

### Response surface plots

Three-dimensional (3D) surface curves and two-dimensional (2D) contour plots were designed to investigate the interactive effects of process factors on *S. aureus* inhibition. In Figs. 2C and D, it was observed that cinnamon played a crucial role in *S. aureus* inhibition. Increased cinnamon led to a higher Inhibition zone diameter because of its thymoquinone components. To validate this prediction, an experiment was conducted, resulting in an experimental value of 5.2 cm, which closely aligned with the predicted IZD (5.2%). The desirability of a model is determined by its proximity to unity that confirms the applicability of the model and the predicted responses.

### Cytotoxicity of EOs mixture against normal HSF cell line

**Fig. 3:**
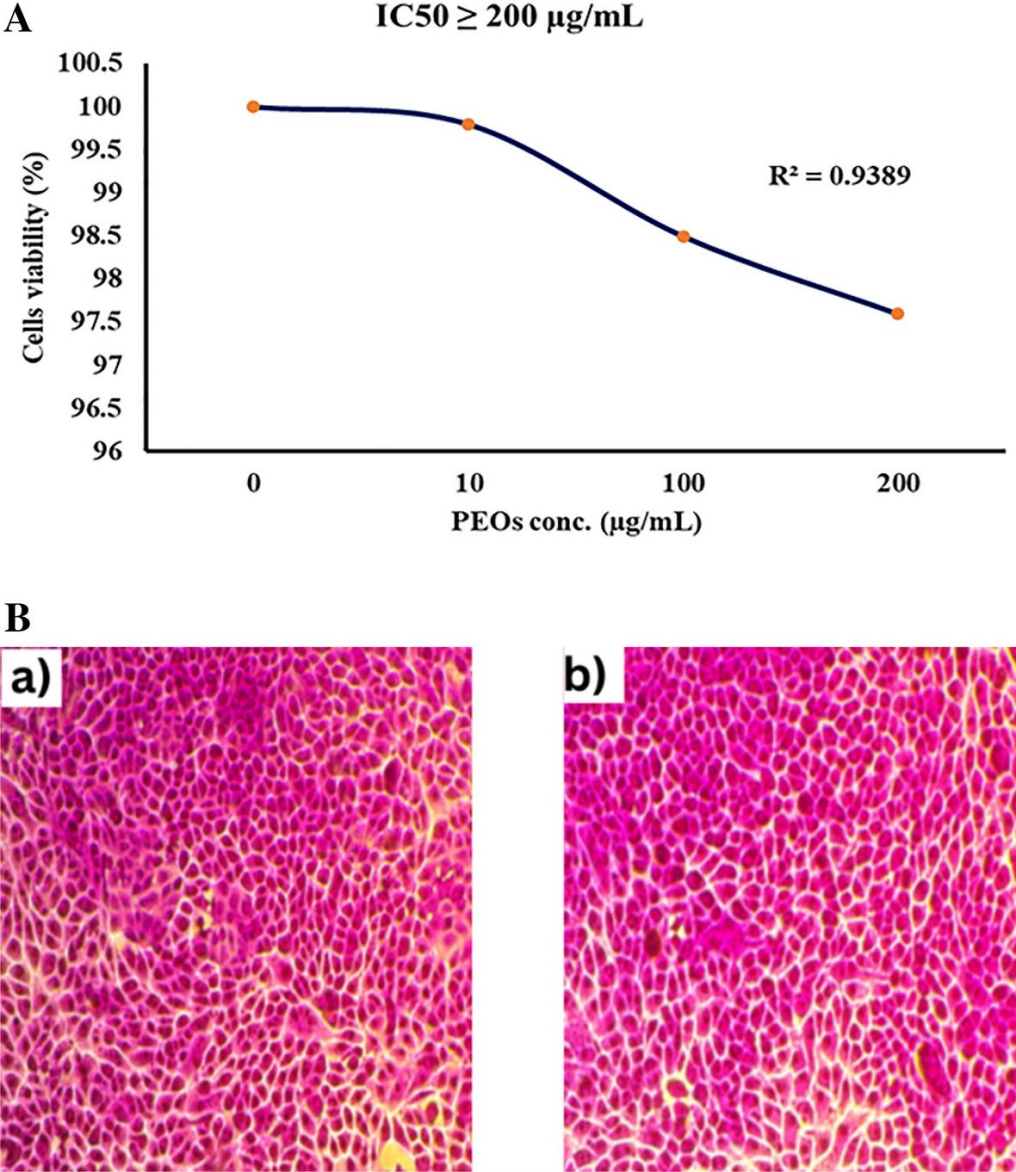
**A** Cell viability (%) of EOs mixture on Normal HSF cell line.** B** Cytotoxicity of moringa, cinnamon, and black seed oil mixture on normal HSF cells maintained in DMEM media supplemented with 100 mg/mL of streptomycin, 100 u/mL of penicillin, and 10% of heat-inactivated fetal bovine serum in humidified 5% (µg/mL) CO_2_ atmosphere incubated at 37 °C (**a**) Control treatment illustrates normal adherent cells; (**b**) *mixture oils* show 97.6% viability

As shown in Fig. 3A and B, cytotoxicity testing showed the optimized essential oil mixture of *Moringa oleifera, Cinnamomum verum, and Nigella sativa* demonstrated minimal adverse effects on normal human skin fibroblast cells at antibacterial concentrations. Recorded cell viabilities remained high at 99.8%, 98.6%, and 97.6% of control cells when treated with 10, 100, and 200 μl/mL of the oil combination over 24 h. The half-maximal inhibitory concentration was greater than 200 μl/mL. Visual inspection also verified maintained cell adherence and morphology following 200 μl/mL oil exposure without appreciable cytotoxic indications. These cytotoxicity results signify excellent biocompatibility of this phytochemical oil formulation, supporting its potential development as a safe antibacterial therapeutic against *S. aureus* pending further preclinical confirmation across extended durations and utilizing additional human cell types.

### Gas chromatographyMass spectrometry (GC/MS) for EOs

As shown in Table 4, Gas chromatography–mass spectrometry analysis characterized the chemical composition of the black seed, moringa, and cinnamon essential oils. Numerous bioactive compounds were identified in each oil. Black seed oil contained high levels (41.75%) of linoleic acid methyl ester, an omega-6 fatty acid with antioxidant and anti-inflammatory activities. Other predominant constituents included the saturated fats palmitic acid methyl ester (12.95%) and methyl stearate (4.50%), as well as cis-11,14-eicosadienoic acid methyl ester (3.18%), an unusual long-chain polyunsaturated fatty acid. Moringa oil was particularly rich (22.62%) in trans-13-octadecenoic acid, a monounsaturated omega-7 fatty acid with medicinal properties. Other major components were the saturated hexadecanoic acid (11.05%) and octadecenoic acid methyl ester (9.52%). An unusual minor constituent was ethyl iso-allocate (6.14%), a bioactive steroid derivative. The most abundant component of cinnamon oil was cinnamaldehyde (24.42%), the signature compound imparting fragrance and flavor. Additionally, sizeable proportions of 3-allyl-6-methoxyphenol (18.55%) and methyl cis-cinnamate (4.43%) were documented, both phenylpropanoids with pharmaceutical effects. Propylene glycol (7.95%) was determined as a major solvent artifact from the extraction process. The diverse spectrum of functional natural products elucidated in the three oils provides insight into their biological and pharmacological activities. Their rich composition underlies potent antibacterial properties.

**Table 4:** Gas chromatography analysis of the adult *Periplaneta americana* cockroach hemolymph indicated the presence of active compounds and fatty acids

Plant oil	Retention time (RT)	Compound name	Area %	Molecular formula
Black seed	25.67	Palmitic Acid methyl ester	12.95	C17H34O2
	28.81	Linoleic Acid methyl ester	41.75	C19H34O2
	29.40	Methyl stearate	4.50	C19H38O2
	32.15	cis-11,14-Eicosadienoic acid, methyl ester	3.18	C21H38O2
	37.81	9,12-Octadecadienoic acid (Z,Z)-, 2-hydroxy-1-(hydroxymethyl)ethyl ester	0.68	C21H38O4
Moringa	25.62	Palmitic Acid methyl ester	3.98	C17H34O2
	26.39	n-Hexadecanoic acid	11.05	C16H32O2
	28.80	9-Octadecenoic acid, methyl ester, (E)-	9.52	C19H36O2
	29.57	trans-13-Octadecenoic acid	22.62	C18H34O2
	44.30	Ethyl iso-allocate	6.14	C26H44O5
Cinnamon	4.7443	Propylene Glycol	7.9458	C3H8O2
	8.0698	Linalool	1.0595	C10H18O
	10.0979	Cinnamaldehyde,(E)-	24.4226	C9H8O
	10.7863	3-Allyl-6-methoxyphenol	18.5486	C10H12O2
	11.051	Methyl cis-cinnamate	4.4267	C10H10O2

## Discussion

This study evaluated the antimicrobial and antibiofilm potential of essential oils from moringa, cinnamon, and black seed against *E.coli*. Susceptibility testing of *S. aureus* indicated resistance against amoxicillin, ampicillin, and azithromycin while exhibiting sensitivity to tetracycline and doxycycline. Similar antibiotic resistance in *S. aureus* isolates has been previously reported, however sensitivity to tetracyclines contrasts with studies showing increased resistance. Understanding factors driving resistance versus sensitivity will inform antibiotic stewardship and evidence-based use of doxycycline as first-line therapy for susceptible *S. aureus* infections (Meshaal et al. 2021; Shah and Mir 2022).

To identify optimal oil combinations, a mixture experimental design was utilized with three variables (moringa, cinnamon, and black seed essential oils), each at three levels (Baj et al. 2023). Polynomial response models revealed that all three oils significantly influenced *S. aureus* inhibition effectiveness. By applying mathematical optimization, the maximal efficacy with an inhibition zone diameter (IZD) of 5.2 cm was achieved with a combination of 100 μl/mL moringa, 125 μl/mL cinnamon, and 100 μl/mL black seed oils. Validation experiments corroborated efficacy predictions. These results identify the rational design of phytochemical oil combinations as an alternative to combat drug-resistant *S. aureus* infections using natural plant products. The current findings align with previous research demonstrating the antimicrobial and antibiofilm properties of moringa (Enan et al. 2020; Lee et al. 2017), cinnamon (Zhang et al. 2016), and black seed (Othman et al. 2018) essential oils individually against *S. aureus.* Our new finding for the synergistic combination of the oils inhibited *S. aureus* growth and biofilm formation, corroborating studies advocating phytochemical mixtures to potentiate therapeutic outcomes (Nemattalab et al. 2022; Rohani et al. 2023). Expanding on those studies, the present work applies response surface methodology to systematically ascertain optimal ratios between key active ingredients in moringa, cinnamon and black seed oils against resistant *S. aureus* isolates. Achieving 5.2 cm inhibition zones rivals antibiotic efficacy (Duarte and Vale 2022). Together, these works provide an impetus for further testing optimized botanical oil formulations as promising, naturally derived therapeutic alternatives to conventional antibiotics for drug-resistant Gram-negative bacteria. Rational phytochemical targeting may enable desensitization and overcome treatment barriers in problematic infections (Jain 2011).

The determined MIC revealed bacterial proliferation was halted with a combination of 0.125 mg/mL moringa, cinnamon, and black seed oils. However, the minimal bactericidal concentration testing demonstrated that 0.0625 mg/mL was incapable of killing *S. aureus* entirely, indicating a bacteriostatic action (Adegbeye et al. 2020; Manilal et al. 2017; Zhang et al. 2016) at or below this concentration. Additional assays examined the impact of the essential oil combination on biofilm establishment. *S. aureus* 24–48-h growth was markedly reduced with 0.5 mg/mL, and further constrained with 0.25 mg/mL exposure. Yet neither concentration fully eliminated biofilm formation, which correlates to the bacteriostatic action. While the oil combination exhibited bacteriostatic qualities, fully preventing *S. aureus* proliferation at 0.125 mg/mL, complete bactericidal action was not achieved even at double that dose based on regrowth after subculturing (Budri et al. 2015). This may explain robust biofilm propagation with sustained low-level exposure, as surviving persistent cells resisted infections. Nonetheless, escalating concentrations did restrict biofilm maturation and thickness, similar to cinnamon oil effects noted against other Gram-negatives. These findings reveal potent bacteriostatic synergy, though complete biofilm eradication may require higher doses, combination with antibiotics, or secondary metabolites targeting resistance mechanisms. Elucidating specific surviving subpopulations could further potentiate natural oil treatment strategies against recurrent device and tissue infections. These findings suggest that higher doses of the essential oil products would be necessary to inactivate existing *S. aureus* biofilms or prevent their new establishment, which merits further investigation for clinical relevance. Another study (Mouwakeh et al. 2019) reported similar dose-dependent impacts from *Nigella sativa* oils on *S. aureus* viability. For clinical relevance, higher concentrations appear necessary to eradicate cells and biofilms as supported by this study. The 0.5 mg/mL oil mixture inhibited but did not eliminate *S. aureus* biofilm formation. Since biofilms confer a 1000-fold increase in antibiotic resistance, exploiting natural products to prevent biofilm establishment has immense therapeutic potential.

The results of this study revealed both synergistic and antagonistic effects among the combinations of moringa, cinnamon, and black seed oils, with the synergy effect (%) ranging from significant synergistic interactions to mild antagonism, despite a consistent FIC index of 2 (Duarte and Vale 2022). This finding aligns with previous studies that have reported variability in the interactions of essential oils when combined, where some combinations enhance antimicrobial efficacy while others exhibit neutral or antagonistic effects. For instance, the strong synergy observed in Run 11 and Run 15, with a synergy effect of 25.6%, is consistent with reports highlighting the potent antimicrobial synergy of cinnamon oil when combined with other oils, likely due to its high cinnamaldehyde content, which disrupts bacterial membranes and enhances the activity of other compounds (Milagres de Almeida et al 2023). On the other hand, the antagonistic effects observed in Run 3, with a synergy effect of − 48.81%, could be attributed to potential competition between the active compounds in the oils, as seen in studies where the combination of certain phytochemicals led to reduced efficacy (Liu 2004; Wang et al 2015). The variability in the results underscores the complexity of essential oil interactions, as factors such as concentration ratios, individual oil composition, and bacterial strain specificity can significantly influence outcomes (Gómez-Llorente et al 2025). These findings suggest that while certain combinations can offer enhanced antimicrobial activity, others may inhibit the effectiveness of the oils, emphasizing the need for careful optimization of concentrations in formulations for therapeutic or preservation purposes.

Cytotoxicity analysis was conducted to evaluate the biocompatibility and preliminary safety profile of the optimized moringa, cinnamon*,* and black seed essential oil combination. Previous results reported no toxic effects for the individual plant oils on different cell lines (Singh et al. 2009; Swamy and Tan 2000). Utilizing the established MTT cell viability assay on normal human skin fibroblast (HSF) cells, minimal adverse effects were demonstrated at doses up to 200 μl/mL oil mixture over 24 h. Recorded viabilities remained high at 99.8%, 98.6%, and 97.6% of control cells at 10, 100, and 200 μl/mL, respectively. The half-maximal inhibitory concentration (IC50) was greater than the maximum tested concentration of 200 μl/mL. These results signify this phytochemical oil mixture holds excellent biocompatibility with human cell lines at the antibacterial concentrations found effective against *Staphylococcus aureus in* preceding experiments. Visual inspection of normal HSF cells validated maintained adherence and morphology following 200 μl/mL oil exposure for 24 h. No apoptosis, detachment, or morphological anomalies were discernible. The absence of cytotoxic indications advocates for the safety and tolerability of this essential oil formulation for therapeutic applications against *S. aureus* and related bacterial strains.

Gas chromatography mass spectrometry on the three essential oils identified the major bioactive constituents in the optimized *E. coli*-inhibiting combination. The predominant compound in black seed oil was linoleic acid methyl ester (41.75%). Thymoquinone is a known key antimicrobial component of black seed oil that likely contributed to its efficacy in tandem with linoleic acid methyl ester (Dera et al. 2021). The palmitic acid methyl ester was the major component for both moringa oil (3.98%) and cinnamon oil (7.9458%). Beyond acting individually against *E. coli*, palmitic acid methyl ester could potentiate the impact of minor constituents identified via synergistic interactions. These include trans-13-Octadecenoic acid from moringa oil and Cinnamaldehyde, (E)-from cinnamon oil (Farag et al. 2022). Elucidating the precise antimicrobial mechanisms underlying the optimized oil formulation warrants investigation to further leverage their therapeutic potential (Abd-Elhalim et al 2023b). Moringa oil's major constituent palmitic acid methyl ester likely contributed to efficacy alongside minor components like trans-13-octadecenoic acid. Cinnamon oil bioactive cinnamaldehyde and eugenol disrupt cell membranes and inhibit enzymatic activity in *S. aureus* and other pathogens (Das et al. 2016). Thymoquinone is the primary antimicrobial phytochemical in black seed oil, but compounds like linoleic acid methyl ester found in high levels herein may provide synergistic enhancement (Dera et al. 2021).

In summary, this work characterized an all-natural synergistic combination of three plant essential oils with proven antimicrobial potential against a multidrug-resistant strain of *E. coli*. Identifying natural products that enhance antibiotic effectiveness could help mitigate over-reliance on synthetic agents accused of propagating resistance. The results advocate for further analysis regarding the clinical adoption of the presented essential oil formulation as a complementary or standalone prophylactic or therapeutic measure for managing drug-resistant *S. aureus* infections (Abd-Elhalim et al 2023a). Follow-up analysis is also justified on the impact of minor phytochemical constituents from the oils on potentiating antimicrobial outcomes beyond the major components identified (Abd-Elhalim et al. 2023b).

In conclusion, this work presents an optimized synergistic essential oil formulation of moringa, cinnamon, and black seed that effectively inhibits the growth of multidrug-resistant *Escherichia coli*. Mathematical modeling identified optimal concentrations of plant essential oils that achieved high antimicrobial efficacy as confirmed by validation experiments. The oil mixture demonstrated similar potency as conventional antibiotics based on inhibition zone diameters. Gas chromatography revealed major bioactive components likely responsible for observed antibacterial outcomes. Encouragingly, cytotoxicity evaluations using the developed oil formulation indicated negligible impacts on normal human cell viability and morphology at doses spanning their antimicrobial range (Abu-Hussien et al. 2022). Collectively, these findings highlight exceptional antimicrobial potency against resistant *S. aureus* with a favorable safety profile from the optimized all-natural plant oil mixture. Results warrant additional research assessing antibiofilm impacts across extended incubation periods as well as efficacy against localized infections (Abu-Hussien et al 2024). Outcomes provide impetus for further analysis related to pharmacological interactions, mechanisms of action, and preclinical development as an alternative or complementary antibacterial prophylactic or therapeutic agent.

## Supplementary Information

Supplementary Material 1.

## Data Availability

The raw data and analyzed data used during the current study are available from the corresponding author upon reasonable request. *S. aureus* isolate was obtained from a previous study and deposited in EMCC culture collection at Microbial resources center (MIRCEN), Cairo, Egypt as *S. aureus* EMCC 1351 and deposited in Genbank with gene accession number OQ766965 (https://www.ncbi.nlm.nih.gov/nuccore/OQ766965).
