# Supplementary material for: Synergistic antimicrobial activity of essential oils mixture of Moringa oleifera, Cinnamomum verum and Nigella sativa against Staphylococcus aureus using L-optimal mixture design
Source: AMB Express. 2025 Jan 28;15:15. doi: 10.1186/s13568-024-01797-y (PMC11775370; doi:10.1186/s13568-024-01797-y)
Supplement: Supplementary file 1 — Supplementary Material 1. [file 13568_2024_1797_MOESM1_ESM.pdf]

Supplementary Material

Synergistic Antimicrobial Activity of Essential oils mixture of *Moringa oleifera*, *Cinnamomum verum* and *Nigella sativa* against *Staphylococcus aureus*

Samah H. Abu-Hussien[1*], Antony R. Nasry[2†], Ziad Samy[2†], Salwa M. El-Sayed[3], Ashraf Bakry[4], Naglaa Ebeed[4], Hesham Elhariry[5], Thanaa ElNoby[6]

[1] Department of Agricultural Microbiology, Faculty of Agriculture, Ain Shams University, Cairo 11241, Egypt.
[2]Biotechnology Program, Faculty of Agriculture, Ain Shams University, Cairo 11241, Egypt.
[3] Department of Agricultural Biochemistry, Faculty of Agriculture, Ain Shams University, Cairo 11241, Egypt.

[4] Department of Genetics, Faculty of Agriculture, Ain Shams University, Cairo 11241, Egypt.

[5] Department of Food Science, Faculty of Agriculture, Ain Shams University, Cairo 11241, Egypt.

[6] Department of Agriculture Economics, Faculty of Agriculture, Ain Shams University, Cairo 11241, Egypt.

*Correspondence:

Samah Abu-Hussien, Samah_hashem1@agr.asu.edu.eg

† Equal contributing authors

Table S1 : Analysis of Variance for Essential Oil mixture against *S. aureus* EMCC1351 inhibition expressed as IZD (cm) using mixture design of RSM

	Source	Sum of Squares	df	Mean Square	F-value	p-value	
**IZD (cm)**	**Model**	21.08	9	2.34	41.75	< 0.0001	significant
	(1)Linear Mixture	0.2530	2	0.1265	2.26	0.1860	
	AB	0.4627	1	0.4627	8.25	0.0283	
	AC	0.0085	1	0.0085	0.1519	0.7102	
	BC	0.0135	1	0.0135	0.2416	0.6405	
	ABC	1.05	1	1.05	18.76	0.0049	
	AB(A-B)	0.0055	1	0.0055	0.0979	0.7649	
	AC(A-C)	0.0352	1	0.0352	0.6282	0.4582	
	BC(B-C)	0.7507	1	0.7507	13.38	0.0106	
	**Residual**	0.3365	6	0.0561			
	Lack of Fit	0.0005	1	0.0005	0.0068	0.9373	not significant

**Std. Dev.**	0.2368	**R²**	0.9843	**Mean**	3.55	**Adjusted R²**	0.9607	**C.V. %**	6.67	**Predicted R²**	0.9623

Table S2: Analysis of Variance for Essential Oil mixture against *S. aureus* inhibition expressed as MIC (µg/mL) using mixture design of RSM

	**Source**	**Sum of Squares**	**df**	**Mean Square**	**F-value**	**p-value**	
**MIC (µg/mL)**	**Model**	0.2124	9	0.0236	156.76	< 0.0001	significant
	[(1)]Linear Mixture	0.0039	2	0.0020	12.99	0.0066	
	AB	0.0146	1	0.0146	96.72	< 0.0001	
	AC	0.0062	1	0.0062	41.37	0.0007	
	BC	0.0037	1	0.0037	24.56	0.0026	
	ABC	0.0008	1	0.0008	5.21	0.0626	
	AB(A-B)	0.0005	1	0.0005	3.38	0.1155	
	AC(A-C)	0.0047	1	0.0047	31.18	0.0014	
	BC(B-C)	0.0257	1	0.0257	170.85	< 0.0001	
	**Residual**	0.0009	6	0.0002			
	Lack of Fit	0.0003	1	0.0003	1.94	0.2227	not significant
	**Std. Dev.** 0.0123	**R^2** 0.9958	**Mean** 0.1309	**Adjusted R^2** 0.9894	**C.V. %** 9.38	**Predicted R^2** 0.8576	
